# ARV-825 Demonstrates Antitumor Activity in Gastric Cancer *via* MYC-Targets and G2M-Checkpoint Signaling Pathways

**DOI:** 10.3389/fonc.2021.753119

**Published:** 2021-10-18

**Authors:** Xinmei Liao, Xiaoqing Qian, Zimu Zhang, Yanfang Tao, Zhiheng Li, Qian Zhang, Hui Liang, Xiaolu Li, Yi Xie, Ran Zhuo, Yanling Chen, You Jiang, Haibo Cao, Jiaqi Niu, Cuili Xue, Jian Ni, Jian Pan, Daxiang Cui

**Affiliations:** ^1^ Institute of Nano Biomedicine and Engineering, Shanghai Engineering Research Centre for Intelligent Diagnosis and Treatment Instrument, Department of Instrument Science and Engineering, School of Electronic Information and Electrical Engineering, Shanghai Jiao Tong University, Shanghai, China; ^2^ School of Biomedical Engineering, Shanghai Jiao Tong University, Shanghai, China; ^3^ Institute of Pediatric Research, Children’s Hospital of Soochow University, Suzhou, China; ^4^ Institute of Nanomedicine, National Engineering Research Centre for Nanotechnology, Shanghai, China

**Keywords:** ARV-825, BRD4, gastric cancer, c-Myc, PLK1

## Abstract

**Objective:**

Suppression of bromodomain and extra terminal (BET) proteins has a bright prospect to treat MYC-driven tumors. Bromodomain containing 4 (BRD4) is one of the BET proteins. ARV-825, consisting of a BRD4 inhibitor conjugated with a cereblon ligand using proteolysis-targeting chimera (PROTAC) technology, was proven to decrease the tumor growth effectively and continuously. Nevertheless, the efficacy and mechanisms of ARV-825 in gastric cancer are still poorly understood.

**Methods:**

Cell counting kit 8 assay, lentivirus infection, Western blotting analysis, Annexin V/propidium iodide (PI) staining, RNA sequencing, a xenograft model, and immunohistochemistry were used to assess the efficacy of ARV-825 in cell level and animal model.

**Results:**

The messenger RNA (mRNA) expression of *BRD*4 in gastric cancer raised significantly than those in normal tissues, which suggested poor outcome of patients with gastric cancer. ARV-825 displayed higher anticancer efficiency in gastric cancer cells than OTX015 and JQ1. ARV-825 could inhibit cell growth, inducing cell cycle block and apoptosis *in vitro*. ARV-825 induced degradation of BRD4, BRD2, BRD3, c-MYC, and polo-like kinase 1 (PLK1) proteins in four gastric cancer cell lines. In addition, cleavage of caspase 3 and poly-ADP-ribose polymerase (PARP) was elevated. Knockdown or overexpression *CRBN* could increase or decrease, respectively, the ARV-825 IC50 of gastric cancer cells. ARV-825 reduced *MYC* and *PLK1* expression in gastric cancer cells. ARV-825 treatment significantly reduced tumor growth without toxic side effects and downregulated the expression of BRD4 *in vivo*.

**Conclusions:**

High mRNA expression of *BRD4* in gastric cancer indicated poor prognosis. ARV-825, a BRD4 inhibitor, could effectively suppress the growth and elevate the apoptosis of gastric cancer cells *via* transcription downregulation of c-MYC and PLK1. These results implied that ARV-825 could be a good therapeutic strategy to treat gastric cancer.

## Introduction

According to the latest World Cancer Report released by the World Health Organization Research Agency for 2020, gastric cancer ranked fifth in the incidence of most common cancers worldwide (1). In 2015, the crude incidence of gastric cancer in China ranked second among common malignant tumors. The mortality rate of gastric cancer was 21.16 per 100,000, in China in 2015, ranking third among malignant tumors (2).

Previous studies have displayed that the onset and development of gastric cancer are intricate processes. At present, the mechanism of gastric cancer remains poorly determined (3, 4). Therefore, a deep insight on the related mechanism of gastric cancer and the search for markers or therapeutic targets with high sensitivity and specificity are useful to improve the quality of life and increase the survival rate of patients with gastric cancer. In order to solve various problems in cancer treatment, many studies also provided direction for our treatment of gastric cancer, such as polarized macrophages, for enhancing tumor targeting and drug sensitivity (5, 6), induced pluripotent stem cells (iPS) enhancing immunotherapy against cancer (7), and review in tumor microenvironment (8).

Bromodomains (BRDs) are protein interaction domains that can identify selectively and bind acetylated histones. The BET proteins (BRD4, BRD3, BRD2, and BRDT) are four important family members of 47 bromodomain-containing proteins (9–11). Aberrant transcription is an index of many diseases. BET proteins have a major part to play in the interaction of transcription complexes with transcription activation. BRD4 is one of the widely studied and important BET proteins in cancer and is generally considered as an epigenetic reader that activated RNA polymerase II to combine active chromatin markers with transcriptional elongation. BRD4 is enriched at *MYC* super enhancer region and activates *MYC* transcription (12, 13).

Therefore, inhibition of BRD4 activity could suppress *MYC* transcription pathway activity and then block the process of cancer development. A series of highly specific inhibitors of BRD4 have been researched and developed. For instance, JQ1 could target tumor-related genes specific for super enhancers and inhibit tumor proliferation and migration in various cancers (14, 15), including gastric cancer (16, 17), breast cancer (18), medulloblastoma (19), pancreatic ductal adenocarcinoma (20), and renal cell carcinoma (21). The BRD4 inhibitor OTX015 is in ongoing phase I clinical trials to treat patients with not only solid tumors but also hematological malignancies and shows a wide range of antitumor activities (22–25).

**Scheme 1:**
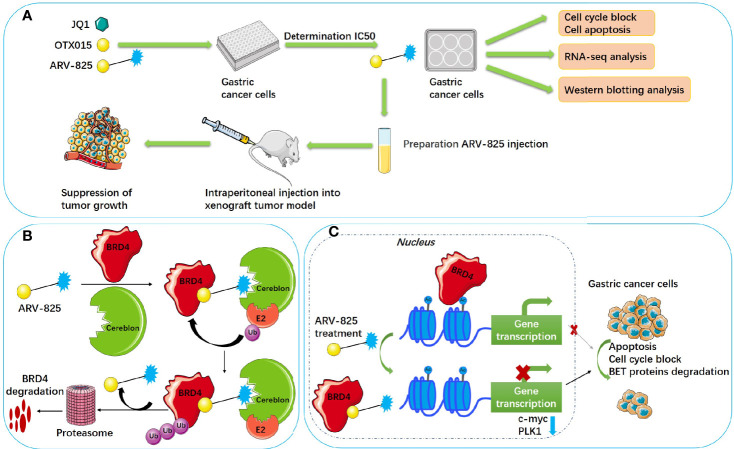
Schematic illustration of ARV-825 treating gastric cancer. **(A)** Technical roadmap of ARV-825 treating gastric cancer. **(B)** Schematic diagram of ARV-825 degrading BRD4. **(C)** Molecular mechanism of ARV-825 treating gastric cancer. Ub, ubiquitin; E2, ubiquitin-conjugating enzyme; cereblon, E3 ubiquitin ligase; Ac, acetylation modification site.

Although previous results indicated that JQ1 and OTX015 have the effect of inhibiting tumors, they also have disadvantages. JQ1 and OTX015 can reaccumulate BRD4 protein and suppress MYC incompletely because of reversibility (26), which results in a higher concentration of the inhibitors being used. To develop better BRD4 inhibitors, proteolysis-targeting chimeras (PROTACs) have emerged (27). PROTACs are blended through a flexible chemical linker combining small molecule drugs with a ligand binding to target proteins; target proteins can be recruited to the ligase and degraded by the ubiquitin–proteasome system (28, 29). ARV-825 consists of OTX015 and the E3 ubiquitin ligase cereblon (CRBN) using PROTAC technology, which degraded BRD4 more efficiently (27). ARV-825 has been studied to treat pancreatic cancer (30, 31), vemurafenib-resistant melanoma (32), cholangiocarcinoma (33), thyroid carcinoma (34), and acute myeloid leukemia (35, 36). ARV-825 could play a critical role in neuroblastoma therapy (37) and T-cell acute lymphoblastic leukemia (38). However, to date, the efficacy of ARV-825 in gastric cancer remains poorly determined. Therefore, the aim of this research is to confirm the antitumor activity and potential mechanisms of ARV-825 against BET proteins in gastric cancer. The effect and mechanisms of ARV-825 treating gastric cancer are shown in **Scheme 1**.

## Methods and Materials

### Cell Culture

The human gastric cancer cell lines, MGC803, HGC27, AGS, SGC7901, BGC823, and SNU-216, were purchase from the cell bank of the Chinese Academy of Science and identified by short tandem repeat analysis within 3 years. Cells were maintained at 37°C with 5% CO_2_. MGC803, HGC27, SGC7901, and BGC823 were cultured in Dulbecco’s modified Eagle’s medium (DMEM) medium; AGS and SNU-216 were cultured in Roswell Park Memorial Institute (RPMI)-1640 medium; medium (Thermo Fisher Scientific) containing 100 U/ml penicillin–streptomycin (Millipore Sigma); and 10% fetal bovine serum (FBS) (Biological Industries).

### Lentivirus Preparation and Infection

*CRBN* was overexpressed in pLX304-CRBN-V5 vector (39). The short hairpin RNA (shRNA) of *CRBN* (the sequences: CCGGGCCCACGAATAGTTGTCATTTCTCGAGAAATGA CAACTATTCG) was constructed in the pLKO.1 vector (40). Envelop plasmid pMD2.G (Cat: 12259, Addgene), packaging plasmid psPAX2 (Cat: 12260, Addgene), and CRBN plasmid were cotransfected into 293FT cells. 293FT cells were transfected for 6 h and cultured with fresh medium for 48 h. The viral supernatant was collected and filtered. Lentiviruses were incubated with gastric cancer cells for 24 h. Puromycin or blasticidin (Sigma-Aldrich) was used to screen for stable cell lines.

### Cell Viability Assay

Gastric cancer cells (1 × 10^4^) were cultured in 96-well plates per well overnight; ARV-825, OTX015, and JQ1 (Cat: HY-16954, HY-15743, HY-13030, MedChemExpress) with different concentrations were added into each well. At 72 h after ARV-825 treatment, CCK8 (Dojindo) was added into 96-well plates per well with previous methods (41). A reader (Thermo Fisher Scientific) read absorbance at 450 nm. Data analysis was conducted by Graph Prism software 8.4.0.

### Clone Formation Assay

MGC803, HGC27, AGS, and SGC7901 cells (1,000–2,000) were cultured in six-well plates per well, respectively. ARV-825 of different concentrations were added to treat cells after 24 h. Incubated with 5% CO_2_ at 37°C for 2 weeks, the gastric cancer cells were fixed with 100% methanol for 15 min and stained with Giemsa for 1 h (Solarbio). The six-well plates were scanned, and the number of clones was counted.

### Cell Cycle Analysis

Gastric cancer cells were collected and centrifugated at 180*g* for 4 min, suspended in cold 70% ethanol overnight, washed with phosphate-buffered saline (PBS), and incubated for 1 h with light-free at room temperature after adding propidium iodide (Cat. P4170, Sigma). The cell cycles were tested by a Beckman Gallios™ Flow Cytometer (Beckman).

### Cell Apoptosis Assay

Cell apoptosis was assessed as previous protocols (42). Gastric cancer cells were treated with ARV-825 of different concentrations. At 72 h after treatment, the cells were trypsinized, centrifugated at 180*g* for 4 min and washed with PBS. The cells were suspended in binding buffer and stained using the fluorescein isothiocyanate-Annexin V apoptosis kit (Cat. 556420, BD Biosciences). The cell apoptosis was tested and analyzed by a Beckman Gallios™ flow cytometer.

### Western Blotting Analysis

Western blotting analysis was carried out as previous protocols (42). Gastric cancer cells were seeded into six-well plates per well; ARV-825 of different concentrations were added to treat cells. After treated with ARV-825 for 72 h, the cells were harvested and extracted by in radioimmunoprecipitation assay (RIPA) buffer. The supernatant was added with loading buffer (Cat. B1012-100, Applygen Technologies) and boiled for 10 min at 98–100°C. Sodium dodecyl sulfate polyacrylamide gel electrophoresis (SDS-PAGE) was performed on proteins and electrotransferred to polyvinylidene fluoride (PVDF) membranes. The membranes were incubated with 5% skimmed milk powder solution and then incubated with primary antibodies at 4°C overnight: anti-BRD4 (Cat. 13440s), anti-BRD2 (Cat. 5848s), anti-poly-ADP-ribose polymerase (PARP) (Cat. 9542), anticleaved-caspase 3 (Cat. 9664), and c-Myc (Cat. 9402) were purchased from Cell Signaling Technology. Other primary antibodies are listed below: anti-BRD3 (Cat. 11859-1-AP, Proteintech), anti-CRBN (Cat. HPA045910, Sigma-Aldrich), anti-polo like kinase 1 (PLK1) (Cat. ab17056, Abcam), and antiglyceraldehyde-3-phosphate dehydrogenase (GAPDH) (MA3374, Millipore). The membranes were incubated with horseradish peroxidase-conjugated (HRP) secondary antibodies: goat antimouse IgG and goat antirabbit IgG (Jackson ImmunoResearch). The immunoreactive proteins were revealed and analyzed using an ECL detection kit (Pierce) and a LAS 4010 imaging system (GE).

### RNA-Sequencing and Analysis

RNA-sequencing (RNA-seq) was implemented using the protocols provided by Novogene (Novogene Co., Ltd.). Total RNA of gastric cancer cells was extracted by the TRIzol^®^ reagent (Invitrogen). First, RNA was reverse transcribed to cDNA for library construction and sequencing. RNA-seq procedure was performed on HGC27 cells treated with ARV-825 (n = 3) or dimethyl sulfoxide (DMSO) (n = 3). Genes of differential expression (|log_2_fold change| > 1 and *p* < 0.05) were identified using Bioconductor limma analysis according to DAVID Bioinformatics Resources v6.8 (https://david.ncifcrf.gov). Gene set enrichment analysis (GSEA) identified a series of genes that were performed to detect cellular pathways that influenced the cell apoptosis induced by ARV-825 according to the GSEA Application (http://www.broadinstitute.org/gsea/).

### *In Vivo* Xenografts Tumor Model

The animal experiments complied with the requirements of the Institutional Animal Care and Use Committee of Shanghai Jiao Tong University (No. 201801054). Four-week-old male nude mice (Lingchang BioTech, n = 6 per group) were injected subcutaneously 5 × 10^6^ HGC27 cells in their front flank. Tumor size was measured every 3 days. The calculation formula of tumor volume is (length × width × height)/2. After 2 weeks, when the size of the engrafted tumor came to about 100 mm^3^, the mice were injected intraperitoneally either ARV-825 at 10 mg/kg or menstruum control (10% Kolliphor^®^HS15, BASF) every day. The mice were sacrificed when the tumors in the control group exceeded 1,000 mm^3^. The tumors were excised and then embedded in paraffin for immunohistochemistry.

### Immunohistochemistry

Immunohistochemistry was carried out as previous protocols (43). The Ki-67 antibody (Cat. ab15580, Abcam) and HRP/DAB detection kit (Cat. ab64261, Abcam) were used. Tissue sections of immunohistochemical staining were examined using the Olympus BX41 imaging system. The calculation formula of the total scoring (TS) was as follows: TS = the intensity (I) × the percentage of positive cells (P).

### Statistical Analysis of Data

SPSS software version 20.0 (IBM) was used to analyze the data. Significant difference of data was analyzed by Student’s t-tests. GraphPad Prism version 8.4.0 was used to draw the figures. In the figures, means ± the standard deviation (SD) is shown; *p*-values < 0.05, the results were statistically significant, for which *p < 0.05, **p < 0.01, and ***p < 0.001.

## Results

### High mRNA Expression of *BRD4* Is Associated With Poor Prognosis in Patients With Gastric Cancer

**Figure 1:**
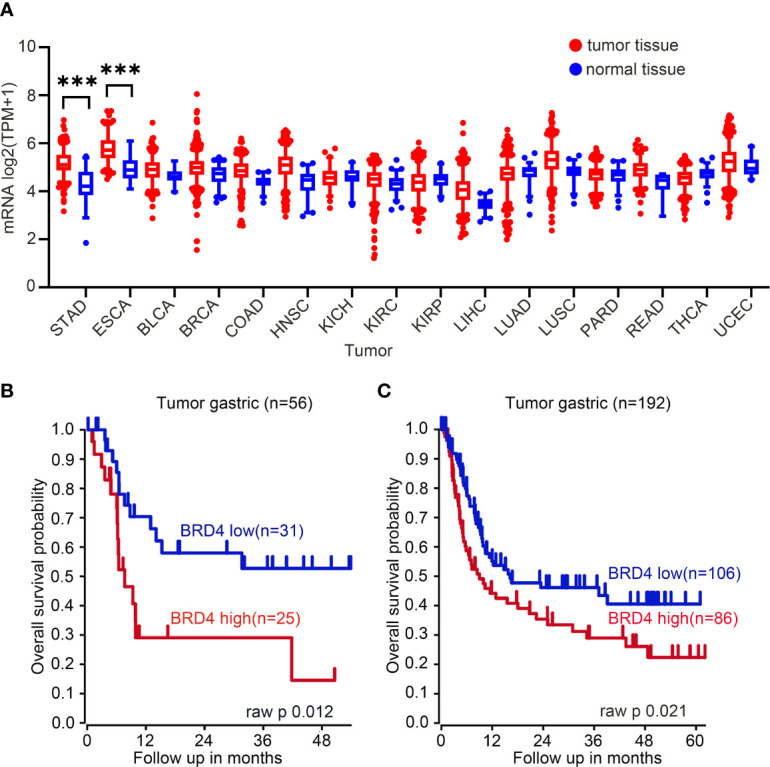
BRD4 mRNA expression in tumor tissues and normal tissues. **(A)** BRD4 mRNA expression in tumor tissues and normal tissues (generated from the web site: http://gepia.cancer-pku.cn/index.html). The mRNA expression of BRD4 in gastric cancer raised significantly than that in normal tissues, there is a similar performance in esophageal adenocarcinoma tissues and normal tissues. STAD, stomach adenocarcinoma; ESCA, esophageal carcinoma; HNSC, head and neck squamous cell carcinoma; KICH, kidney chromophobe; BLCA, bladder urothelial carcinoma; KIRC, kidney renal clear cell carcinoma; UCEC, uterine corpus endometrial carcinoma; THCA, thyroid carcinoma; COAD, colon adenocarcinoma; LUSC, lung squamous cell carcinoma; KIRP, kidney renal papillary cell carcinoma; READ: rectum adenocarcinoma; BRCA: breast invasive carcinoma; PRAD, prostate adenocarcinoma; LUAD, lung adenocarcinoma; LIHC, liver hepatocellular carcinoma. **(B)** Overall survival curve including 56 patients with gastric cancer. Kaplan–Meier curves were generated from gastric tumor (Tan-56-fRMA-u133p2; source: GEO ID, gse34942). **(C)** Overall survival curve including 192 patients with gastric cancer (Tan-192-fRMA-u133p2; source: GEO ID, gse15459). Survival curve data originated from R2 Platform (http://r2.amc.nl). The cutoff point of high or low BRD4 expression was median survival time. ***p < 0.001.

Expression of *BRD4* was measured in different kinds of tumors, which was displayed using gene expression profiling interactive analysis (GEPIA, http://gepia.cancer-pku.cn/index.html), which illustrated that mRNA expression of *BRD4* in tumor tissue was raised remarkably than that in normal tissue only in stomach adenocarcinoma (STAD) and esophageal adenocarcinoma (ESCA, **Figure 1A**). High mRNA expression of *BRD4* was associated with poor overall survival of patients with gastric cancer (**Figures 1B, C**). The criterion of categorizing high or low expression was median survival time.

### ARV-825 Suppresses Cell Viability of Gastric Cancer Cells

**Figure 2:**
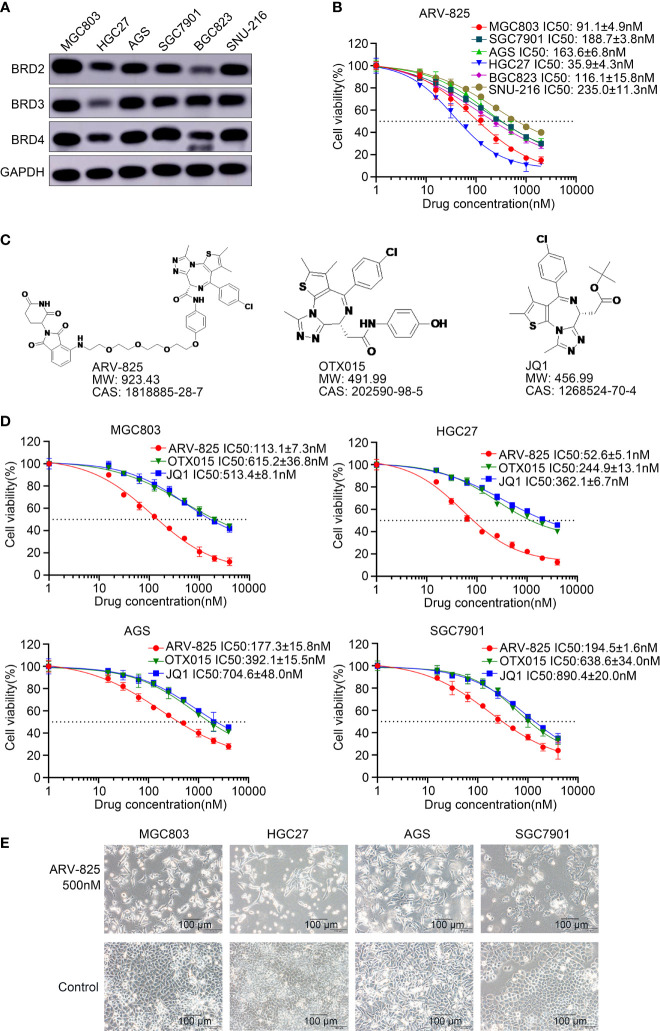
ARV-825 suppresses the viability of gastric cancer cells. **(A)** BET protein levels in gastric cancer cells. **(B)** Viability of gastric cancer cells treated with ARV-825 at different concentrations. Values of IC50 have been calculated and shown on the graph. **(C)** Chemical structures of ARV-825, OTX015, and JQ1. **(D)** The cell viability of MGC803, HGC27, AGS, and SGC7901 cells treated with ARV-825, OTX015, and JQ1. **(E)** Morphology of MGC803, HGC27, AGS, and SGC7901 cells treated with ARV-825.

**Figure 3:**
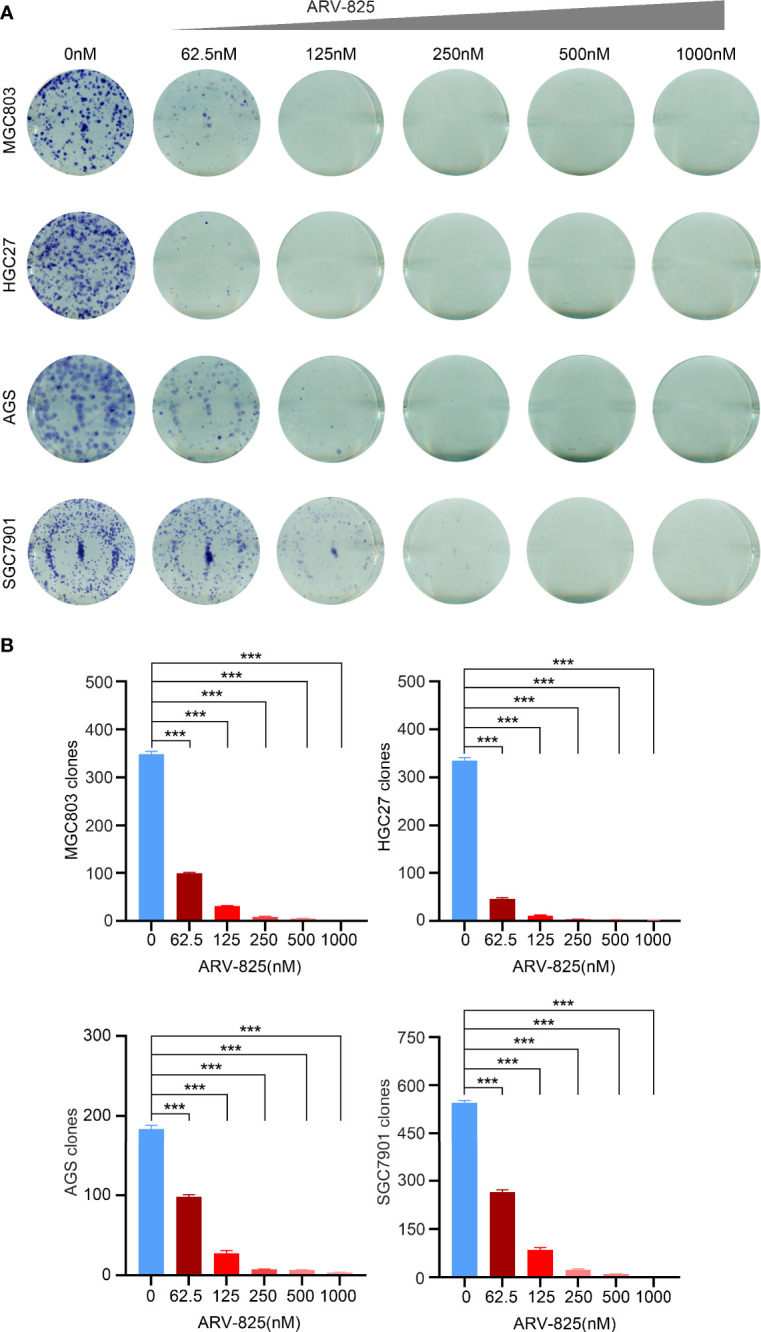
ARV-825 suppresses clonal formation of gastric cancer cells. **(A)** ARV-825 suppressed dose dependently the clonal formation of gastric cancer cells. **(B)** Clone numbers of gastric cancer cells had remarkable difference between the ARV-825 treatment group and the control group. ***p < 0.001.

BRD4, BRD2, and BRD3 were abundantly expressed in MGC803, HGC27, AGS, SGC7901, BGC823, and SNU-216 cells (**Figure 2A**), implying that the BET proteins were diffusely expressed in gastric cancer cells. ARV-825 consists of CRBN and OTX015. The effect of increasing doses of ARV-825 on gastric cancer cell lines incubated for 72 h was evaluated. CCK8 assays showed that a dose-dependent decrease in gastric cancer cell viability was observed after ARV-825 treatment (**Figure 2B**). The chemical structures of ARV-825, OTX015, and JQ1 are shown in **Figure 2C**. HGC27 and MGC803 had lower IC50 than other gastric cancer cells. The IC50 values of other BRD4 inhibitors, such as OTX015 and JQ1, were compared with that of ARV-825 in gastric cancer cells (**Figure 2D**). The results showed that ARV-825 had lower IC50 values and showed a better suppression effect on gastric cancer cell viability than OTX015 and JQ1. Decreased quantity and shrinkage of the volume of gastric cancer cell were examined in the group treated with ARV-825 (**Figure 2E**), compared with that in the untreated control group. Clonal formation assay was applied to observe the influence of ARV-825 on the long-term growth of gastric cancer cells (**Figure 3A**); ARV-825 suppressed dose dependently the clonal formation of MGC803, HGC27, AGS, and SGC7901 cells. In conclusion, these findings showed that ARV-825 had antiproliferative effect in gastric cancer cells; the number of clones of ARV-825-treatment groups was remarkably lower compared with that of control groups (**Figure 3B**).

### ARV-825 Induces the Degradation of BRD4 Related to CRBN

**Figure 4:**
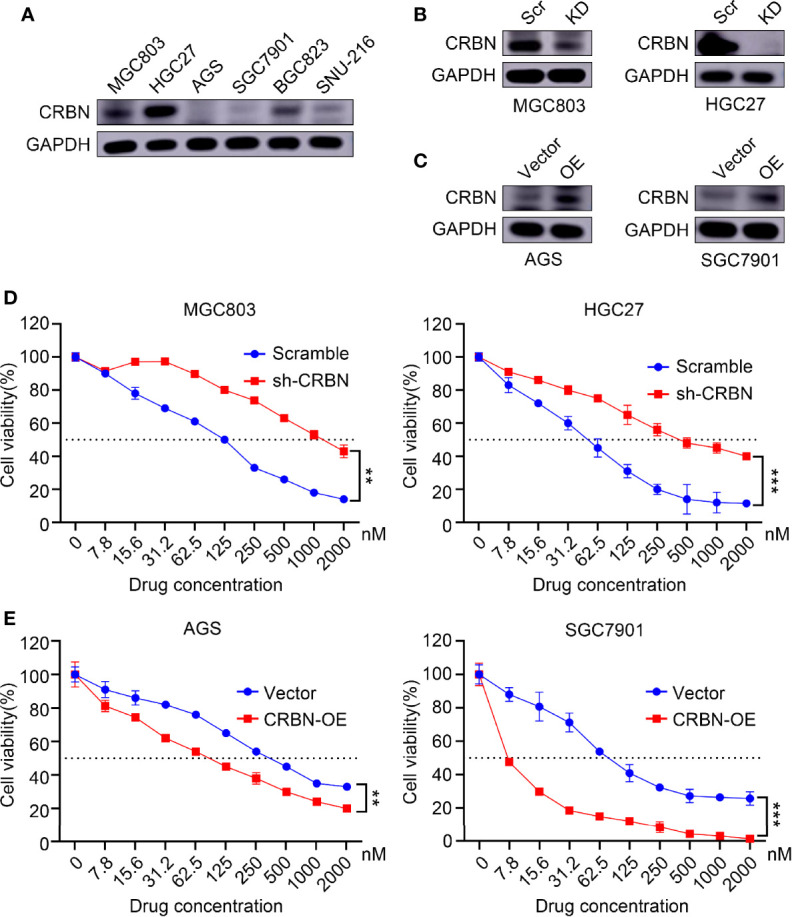
CRBN performs an important role in response to ARV-825 in gastric cancer cells. **(A)** CRBN protein levels in gastric cancer cells by Western blotting analysis. **(B)** Western blotting analysis showing CRBN protein level after knockdown CRBN by sh-CRBN lentivirus in MGC803 cells and HGC27 cells. KD, knockdown, Scr, Scramble. **(C)** Western blotting analysis showing CRBN protein level after overexpressing CRBN in AGS cells and SGC7901 cells. OE, overexpression. **(D)** Cells of knockdown CRBN increased cell viability after treated with ARV-825 in MGC803 cells and HGC27 cells. **(E)** Cells of overexpressing CRBN decreased cell viability after treated with ARV-825 in AGS cells and SGC7901 cells. **p < 0.01, ***p < 0.001.

MGC803, HGC27, and BGC823 express substantial amounts of CRBN, while AGS, SGC7901, and SNU-216 cells have relatively low expression of CRBN (**Figure 4A**). The effect of ARV-825 in gastric cancer cells is associated with CRBN expression. Knockdown of *CRBN* expression in MGC803 and HGC27 cells could decrease partly the growth inhibition influence of ARV-825 (**Figures 4B, D**). By contrast, overexpression of *CRBN* in gastric cancer cells significantly increased the sensitivity of AGS and SGC7901 cells to ARV-825 (**Figures 4C, E**). These results suggested that CRBN is associated with the growth inhibition activity of ARV-825.

### ARV-825 Induces Cell Cycle Block and Apoptosis in Gastric Cancer Cells

**Figure 5:**
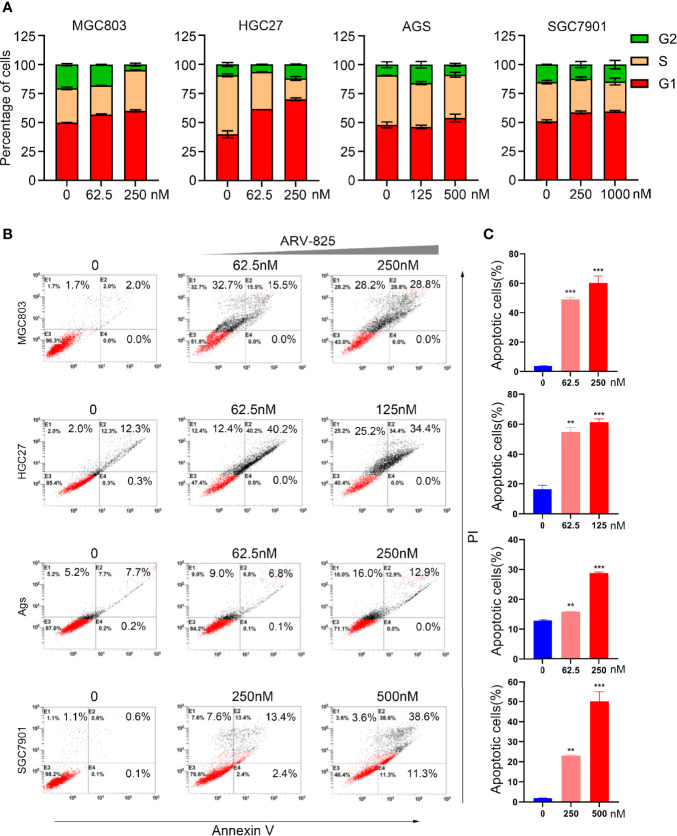
ARV-825 induces cell cycle block and apoptosis of gastric cancer cells. **(A)** ARV-825 increased ratio of G1 phase cells and simultaneously decreased the ratio of S phase and G2 phase cells in MGC803, HGC27, AGS, and SGC7901 cells *via* cell cycle analysis. **(B)** The ratio of apoptotic cells among gastric cancer cells increased dose dependently after treated with ARV-825 *via* cell apoptosis analysis. **(C)** The ratio of apoptotic cells markedly elevated dose dependently in gastric cancer cells with ARV-825 treatment. **p < 0.01, ***p < 0.001.

**Figure 6:**
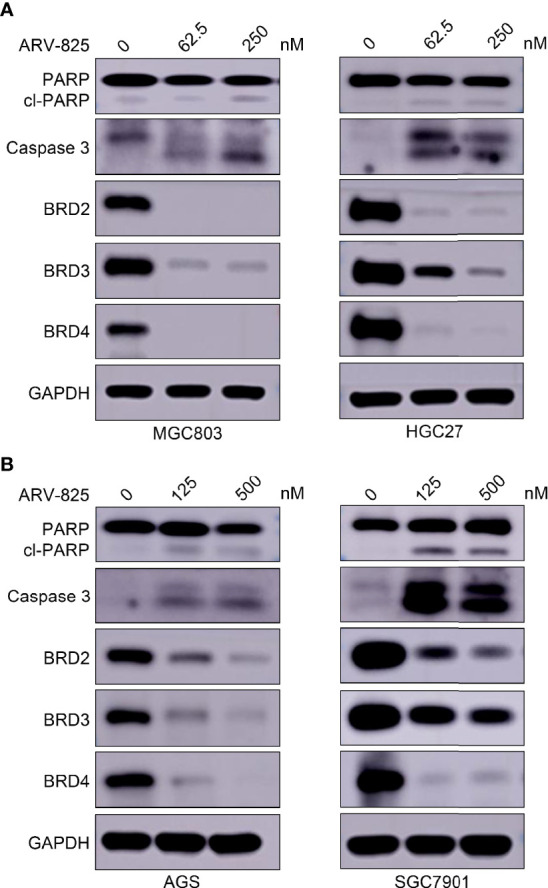
ARV-825 degrades BET proteins in gastric cancer cells. **(A)** ARV-825 robustly degraded BET protein and induced caspase 3 and PARP cleavage in MGC803 and HGC27 cells. **(B)** ARV-825 robustly degraded BET protein and induced caspase 3 and PARP cleavage in AGS and SGC7901 cells.

We investigated whether ARV-825 could regulate the cell cycle in gastric cancer cells. MGC803, HGC27, AGS, and SGC7901 cells were treated with ARV-825 for 24 h to perform cell cycle analysis. Compared with the control group, the ARV-825 treatment group showed an increase in the ratio of G1 phase cells and a reduction in the ratio of G2 and S phases cells simultaneously (**Figure 5A**). Apoptosis of gastric cancer cells was also examined after ARV-825 treatment. The Annexin V/PI staining analysis demonstrated that ARV-825 increased cell apoptosis, presenting dose dependence. The ratio of apoptotic cells in the groups with ARV-825 treatment increased dose dependently in contrast to control groups (**Figures 5B, C**). Western blotting analysis revealed that ARV-825 could increase activation of PARP and caspase-3 in the four gastric cancer cell lines (**Figures 6A, B**). These results demonstrated that ARV-825 could induce cell cycle block and apoptosis of gastric cancer cells.

### ARV-825 Degrades BET Proteins in Gastric Cancer Cells

ARV-825 robustly degrades BRD4 protein *via* the ubiquitin–proteasome system, which consists of a CRBN-recruiting moiety and OTX015. Therefore, we observed the BET protein levels in gastric cancer cells with ARV-825 treatment. Western blotting analysis illustrated that ARV-825 displayed efficient degradation of BRD4 in four gastric cancer cells (**Figures 6A, B**). In addition, ARV-825 could decrease simultaneously BRD2 and BRD3 (**Figures 6A, B**). Gastric cancer cells treated with ARV-825 also resulted in PARP and caspase 3 cleavage. The experimental progress indicated that ARV-825 could downregulate expression level of BET protein in gastric cancer cells.

### ARV-825 Downregulates *MYC* and *PLK1* Expression in Gastric Cancer Cells

**Figure 7:**
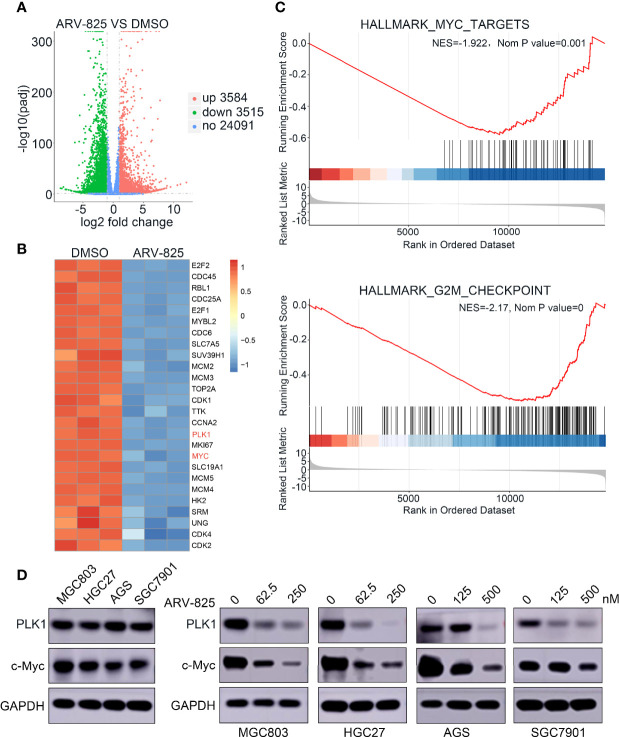
ARV-825 decreases MYC and PLK1 expression in gastric cancer cells. **(A)** Volcano plot analysis of the RNA-seq illustrated expression changes of genes in HGC27 cells between ARV-825 treatment group and the control group. Genes highlighted in red were upregulated, and those in blue were downregulated; black indicates unchanged expression. **(B)** Heat-map view displayed the genes of differential expression in HGC27 cells treated with 125 nM ARV-825 (|log_2_fold change> 1, *p* < 0.05); these genes included c-Myc and PLK1 targets. Each column indicates a sample; each row indicates a gene, The color changes with different expression of each gene. Blue represents downregulation; red represents upregulation. **(C)** GSEA plots displayed gene enrichment in HALLMARK_MYC_TARGETS and HALLMARK_G2M_CHECKPOINT signaling pathways in HGC27 cells treated with ARV-825. **(D)** PLK1 and c-Myc protein levels were decreased by ARV-825 treatment in MGC803, HGC27, AGS, and SGC7901 cells.

To determine the potential mechanism of ARV-825, RNA-seq was used to screen and analyze genes (GEO ID: GSE179581). As shown in **Figure 7A**, under the condition of |log_2_fold change | > 1 and an adjusted *p* < 0.05, compared with those in the control group, 3,584 genes were identified as upregulated and 3,515 genes were downregulated in HGC27 cells of ARV-825 treatment. We continued to analyze the signaling pathways and identify associated genes. ARV-825 markedly downregulated the expression levels of *E2F2*, *CDC45*, *RBL1*, *CDC25A*, *CDK1*, *PLK1*, *MYC*, *SLC19A1*, *MCM5*, *MCM4*, *HK2*, *SRM*, *UNG*, *CDK4*, and *CDK2* (**Figure 7B**). All the significant GSEA hallmarks have been shown in **Supplementary Table S1**; HALLMARK_G2M_CHECKPOINT and HALLMARK_MYC_TARGETS, which are related with cell-cycle functions, are the top 5 negative hallmarks. GSEA plots showed gene enrichment in HALLMARK_G2M_CHECKPOINT and HALLMARK_MYC_TARGETS signaling pathways after ARV-825 treatment in HGC27 cells. Many genes in the G2M_CHECKPOINT and MYC_TARGETS signaling pathways were downregulated (**Figure 7C**), *MYC* and *PLK1* genes were both downregulated in these two signaling pathways. As expected, Western blotting analysis confirmed that c-Myc and PLK1 protein levels decreased dose dependently with ARV-825 treatment in MGC803, HGC27, AGS, and SGC7901 cells (**Figure 7D**). These results revealed that ARV-825 disturbed BRD4-mediated *MYC* and *PLK1* transcription, resulting in decreasing c-Myc and PLK1 protein levels.

### ARV-825 Suppresses Tumor Growth in The Xenograft Tumor Model

**Figure 8:**
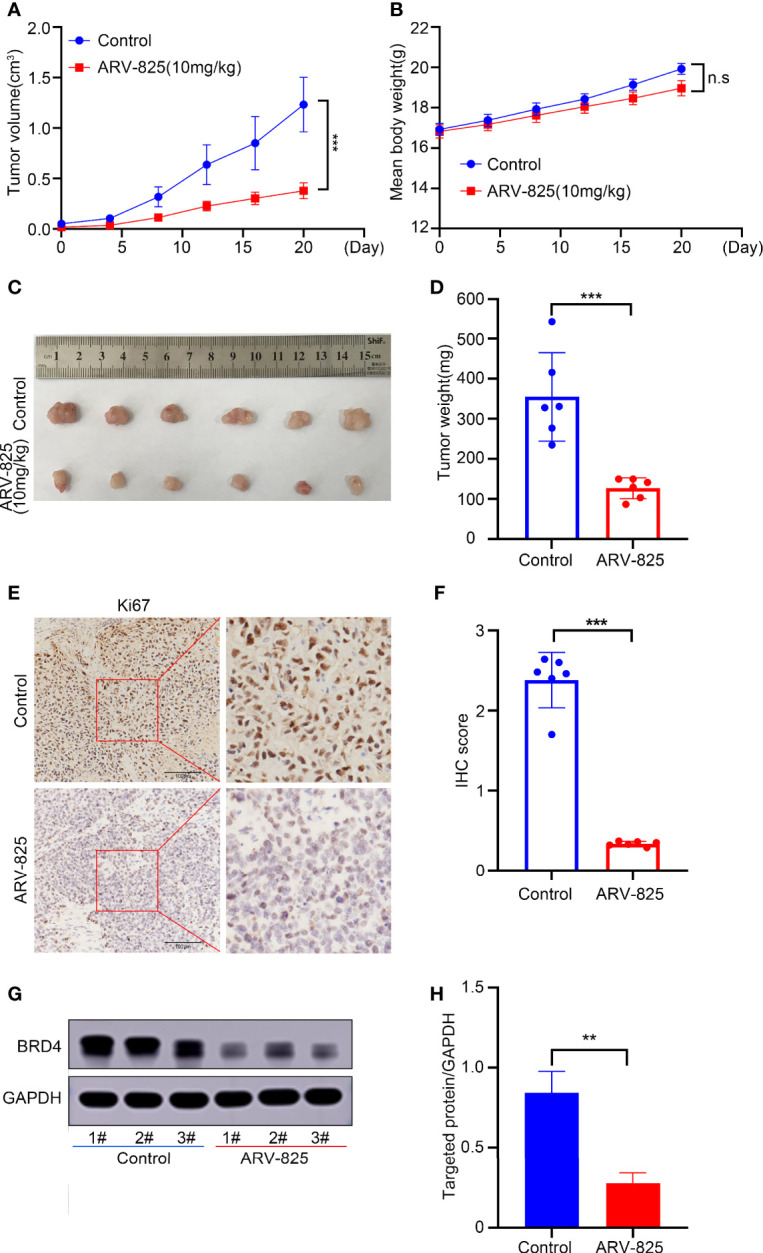
ARV-825 suppresses tumor growth in the xenograft tumor model. Nude mice (n = 6) of HGC27 xenograft tumor were injected intraperitoneally by 10 mg/kg ARV-825 or menstruum control (10% Kolliphor^®^HS15) daily for 20 days. **(A)** Changes in the tumor volume in mice treated with ARV-825 and menstruum control. **(B)** The weight of mice was monitored during the experiment. **(C)** Pictures of xenograft tumors from mice treated with ARV-825 and menstruum control. **(D)** Tumor weight from mice treated with ARV-825 and menstruum control. **(E)** Immunohistochemical staining of Ki67 from xenograft tumors. **(F)** Immunohistochemical scoring of Ki67 staining from xenograft tumors. The total scoring (TS) = the intensity (I) × percentage of positive cells (P). **(G)** BRD4 protein levels in xenograft tumors. **(H)** ARV-825 inhibited BRD4 protein levels from xenografted tumors. n.s, not significant, **p < 0.01; ***p < 0.001.

To study the antitumor effect of ARV-825 *in vivo*, a xenograft model of gastric cancer using HGC27 cells was established. ARV-825 at 10 mg/kg was intraperitoneally injected into mice daily when the volume of subcutaneous tumor achieved about 100 mm^3^. The tumor burden of the ARV-825 treatment group was significantly reduced (**Figures 8A, C, D**) in contrast to that in the control group.

Meanwhile, the treatment group and control group had no remarkable difference in mouse body weight (**Figure 8B**). Immunohistochemical analysis showed that the ratio of Ki67-positive cells was markedly lower in tumors with ARV-825 treatment than that in the control group (**Figures 8E, F**), illustrating the tumor-inhibiting effect of ARV-825. Furthermore, the levels of BRD4 protein were downregulated significantly by ARV-825 treatment *in vivo* (**Figures 8G, H**). These results indicated that ARV-825 could remarkably suppress the tumor growth of gastric cancer without obvious side effects.

## Discussion

Gastric cancer has a high incidence rate and high mortality rate in China (44). The mechanism of gastric cancer remains poorly understood (3, 4). Treatment of advanced gastric cancer remains a challenge, especially for patients with drug insensitivity or metastasis. For this reason, it is urgent and essential to understand the mechanism of the occurrence and development of gastric cancer and find drug targets to treat gastric cancer.

In 2013, Young’s laboratory defined the super enhancer (SE), based on research into enhancers in embryonic stem cells (45, 46). Super enhancers are 8–20 kb long cis-acting elements with transcriptional enhancement activity, which can enrich the density of master transcription factors, cofactors, and histone modification marks. They also activate the expression of identity determining genes in stem cells and have a major part to play in regulating cell fate. The expression of super enhancer-related genes is more easily affected by transcriptional interference; therefore, the application of transcriptional interference agents in tumor cells might be an effective strategy to treat tumors and develop new drugs. As protein interaction domains, bromodomains (BRDs) can selectively identify and bind acetylated histones in the super enhancer region. The BET proteins (BRD4, BRD3, BRD2, and BRDT) can read acetyl-lysine residues of histones and have a major part to play in transcriptional elongation (9–11). Inhibitors (such as JQ1) targeting BRD4 can target tumor-specific super enhancer-related genes in various kinds of tumors and suppress tumor proliferation and migration (14, 15).

The effectiveness of JQ1 has been reported in many previous studies (16–21), including those of gastric cancer (16, 17). OTX015, another BRD4 inhibitor, shows a wide range of antitumor activities (22–25). The actions of OTX015 and JQ1 are reversible, resulting in partial suppression of MYC and the reaccumulation of BRD4 protein (26). To address the limitations of OTX015 and JQ1, proteolysis targeting chimeras (PROTAC) have emerged. ARV-825 consists of OTX015 and the E3 ubiquitin ligase cereblon (CRBN) used in PROTAC technology *via* a flexible chemical linker, which could efficiently degrade BRD4 (27).

ARV-825 has been studied to treat pancreatic cancer (30, 31), vemurafenib-resistant melanoma (32), cholangiocarcinoma (33), thyroid carcinoma (34), acute myeloid leukemia (35, 36, 38), and neuroblastoma therapy (37). These studies demonstrated that ARV-825 had a more effective inhibition on BRD4 protein. In this study, ARV-825 in gastric cancer had lower IC50 than that of JQ1 and OTX015, more thorough degradation of BRD4 and less toxicity and side effects *in vivo*. ARV-825 could effectively degrade BRD2 and BRD3 beside BRD4 *in vitro* and *in vivo*. Similar findings were revealed in other studies using BETi and OTX015 treatment (40, 47). Presumably, the reason is that BET family members have highly homologous domains (48). Further study is needed to determine mechanism of BET inhibitors. As an E3 ubiquitin ligase, CRBN (cereblon) can recruit target proteins and efficiently boost the degradation of target proteins *via* the ubiquitin–proteasome system. Our results revealed that CRBN expression had a major part to play in the inhibition of ARV-825 in gastric cancer cells; knockdown *CRBN* could decrease the inhibition of ARV-825 in MGC803 and HGC27 cells. Correspondingly, CRBN overexpression raised the inhibition of ARV-825 in AGS and SGC7901 cells. Particularly, the sensitivity of SGC7901 cells to ARV-825 increased dramatically. These findings were aligned with a previous research that the CRBN expression level was regarded as an indicator of ARV-825 efficacy (49).

RNA-seq and Western blotting analysis showed how ARV-825 influenced gene expression in gastric cancer cells. The findings demonstrated that inhibiting BRD4 by ARV-825 led to an expression reduction in MYC and PLK1 at mRNA and protein levels in gastric cancer cells. Ba Mingchen et al. also described that BRD4 could boost the growth of gastric cancer cells by activating c-Myc signaling pathway (50). GSEA plots showed the enrichment of genes in signaling pathways after ARV-825 treatment of HGC27 cells. C-Myc and PLK1 were both downregulated in the MYC_TARGETS and G2M_CHECKPOINT signaling pathways. Wu et al. (38) reported that ARV-825 inhibited T-cell acute lymphoblastic leukemia by Myc-pathway genes; RNA-Seq data in this paper also revealed negative hallmark_myc_targets, which was verified by our research results in gastric cancer.

Thousands of up- and downregulated genes were identified by RNA-seq analysis, among which *MYC* and *PLK1* were confirmed as downregulated makers after treatment with ARV-825 in gastric cancer cells. This research found that *PLK1* is an important target gene in gastric cancer in addition to *MYC*. *PLK1* is a good target for many tumors. Cai et al. revealed that high expression of *PLK1* in gastric cancer cells augmented the metastatic ability of tumor cells (51). Otsu et al. reported that patients had poor recurrence-free survival in the case of high *PLK1* expression and DNA aneuploidy (52). Dang et al. analyzed tumorigenesis and investigated whether BI6727 (an inhibitor of PLK1) could effectively inhibit growth of the tumors (53). ARV-825 may have a good antitumor effect on PLK1 high expression tumor; how ARV-825 downregulate *PLK1* is our subject in the following study. Further investigation of the RNA-seq data in gastric cancer will likely identify new drug targets and important signaling pathways. Many other genes are still waiting to be discovered.

In the xenograft model, ARV-825 suppressed the xenograft tumor growth of HGC27 cells. ARV-825 could downregulate BRD4 protein level *in vivo* in consistence with results *in vitro*. This further verified that ARV-825 could block BRD4-MYCN pathway effectively. It also showed that body weight gain had no statistically significance between mice treated with ARV-825 and the control group. Other obvious side effect was not detected in organs from mice with ARV-825 treatment. A recent report has indicated that mice with JQ1 treatment had a decrease in body weight because of impaired adipogenesis capability (54). All of these results states clearly that ARV-825 has good efficacy and is safety.

## Conclusions

High expression of *BRD4* indicated poor prognosis in patients with gastric cancer. ARV-825, a BRD4 inhibitor, could effectively suppress the growth and elevate the apoptosis of gastric cancer cells *via* transcription downregulation of c-MYC and PLK1. ARV-825 in gastric cancer had lower IC50, more thorough degradation of BRD4, and less toxicity and side effects *in vivo*. These results implied that ARV-825 could be a good therapeutic strategy to treat gastric cancer.

## Data Availability Statement

The datasets presented in this study can be found in online repositories. The names of the repository/repositories and accession number(s) can be found below: https://www.ncbi.nlm.nih.gov/, GSE179581.

## Ethics Statement

The animal study was reviewed and approved by the Institutional Animal Care and Use Committee of Shanghai Jiao Tong University.

## Author Contributions

XL, JP, DC, and JNi designed and directed the study. XL, XQ, and ZZ conducted the experiments and wrote the paper. YT and ZL helped with statistical analysis. QZ and HL helped with some of the experiments. ZZ and RZ participated in the *in vivo* experiments. HC, XiaL, XinL, and YX performed the lentivirus preparation and transfection. YC and YJ helped with flow-cytometric analysis, JNiu and CX conducted public data collection and analysis. All authors contributed to the article and approved the submitted version.

## Funding

This work was supported by grants from the National Key Research and Development Program of China (Nos. 2017YFA0205301 and 2017YFA0205304), the National Natural Science Foundation of China (No. 81903169, No. 82072767, No. 81770145, and No. 81602192), Shanghai Sailing Program (No. 19YF1422300), Natural Science Foundation of Jiangsu Province (No. BK20191175, No. BK20190185), Gusu Health Talents program of Soochow City (No. 2020-104), and the Applied Foundational Research of Medical and Health Care of Suzhou City (No. SYS201907).

## Conflict of Interest

The authors declare that the research was conducted in the absence of any commercial or financial relationships that could be construed as a potential conflict of interest.

## Publisher’s Note

All claims expressed in this article are solely those of the authors and do not necessarily represent those of their affiliated organizations, or those of the publisher, the editors and the reviewers. Any product that may be evaluated in this article, or claim that may be made by its manufacturer, is not guaranteed or endorsed by the publisher.
